# Item response analysis of the Geriatric Anxiety Inventory among the elderly in China: dimensionality and differential item functioning test

**DOI:** 10.1186/s12877-019-1346-1

**Published:** 2019-11-15

**Authors:** Zhongquan Li, Xia Zhao, Ang Sheng, Li Wang

**Affiliations:** 1 School of Social and Behavioral Sciences, Nanjing University, 163 Xianlin Avenue, Qixia District, Nanjing, 210023 Jiangsu China; 2 Laboratory for Traumatic Stress Studies, CAS Key Laboratory of Mental Health, Institute of Psychology, Chinese Academy of Sciences, Beijing, China; 3 Department of Psychology, University of Chinese Academy of Sciences, Beijing, China

**Keywords:** Geriatric anxiety inventory, Mokken scale technique, Differential item functioning, Dimensionality

## Abstract

**Background:**

Anxiety symptoms are pervasive among elderly populations around the world. The Geriatric Anxiety Inventory (the GAI) has been developed and widely used in screening those suffering from severe symptoms. Although debates about its dimensionality have been mostly resolved by Molde et al. (2019) with bifactor modeling, evidence regarding its measurement invariance across sex and somatic diseases is still missing.

**Methods:**

This study attempted to provide complemental evidence to the dimensionality debates of the GAI with Mokken scale analysis and to examine its measurement invariance across sex and somatic diseases by conducting differential item functioning (DIF) analysis among a sample of older Chinese adults. The data was from responses of a large representative sample (*N* = 1314) in the Chinese National Survey Data Archive, focusing on the mental health of elderly adults.

**Results:**

The results of Mokken scale analysis confirmed the unidimensionality of the GAI, and DIF analysis indicated measurement invariance of this inventory across individuals with different sex and somatic diseases, with just a few items exhibiting item bias but all of them negligible.

**Conclusions:**

All these findings supported the use of this inventory among Chinese elders to screen anxiety symptoms and to make comparisons across sex and somatic diseases.

## Background

As the geriatric population increases, mental health of the elderly gains more and more substantial concerns, such as depression and anxiety. Prevalence estimates of anxiety disorders ranged from 3.2 to 14.2% in Switzerland and France, as reported in a comprehensive review of geriatric anxiety disorders [1]. Moreover, a survey in one city in China, Chongqing, indicated that 21.63% of older people suffered anxiety, especially among those with physical illness [2]. Though anxiety disorders are highly prevalent among older adults, screening instruments for the aged leave much to be desired [3]. Besides confusion with other disorders [4], cognitive deficits and somatic symptoms account together for the unsatisfactory validity of most measures [5, 6]. To overcome the above deficiencies, Pachana et al. developed the Geriatric Anxiety Inventory (GAI), especially for older populations [3].

The Geriatric Anxiety Inventory only has 20 brief items and facilitates studies regarding anxiety disorders of the elderly prominently. It features a dichotomous and single direction response format, which can decrease the cognitive load of respondents. It also involves minimal somatic symptoms, which helps distinguish mental disorders from somatic diseases [3]. Numerous studies have provided strong evidence for its desirability, with internal consistency ranging from 0.91 to 0.95 [3, 7], test-retest reliability ranging from 0.91–0.99 [3, 8] and good convergent validity [3, 9]. For probing DSM-IV Generalized Anxiety Disorder (GAD), a cut-point of 10/11 in the Geriatric Anxiety Inventory had a specificity of 84% and a sensitivity of 75, and 83% of patients were correctly classified [3]. In another study, an optimal cutoff of 9 was suggested, which had a 100% sensitivity and a 60% specificity, with 65% of participants correctly classified [10]. In short, the psychometric properties of GAI were proven to be excellent, which made it a promising screening and assessment of anxiety among the elderly.

Factor structure is essential in understanding, scoring, and interpreting the responses on the GAI [11]. The GAI was developed as a measure of a unidimensional construct [3, 12]. However, researchers have not reached a consensus on the factor structure of this instrument. The one-factor model was confirmed by Johnco et al. among 256 community-dwelling old adults in Australia [13], among older people living in Beijing communities [14] and among institutionalized old population in Portugal using both exploratory factor analysis (EFA) and confirmatory factor analysis (CFA) [15]. The unidimensionality was further supported by Molde et al. among psychogeriatric mixed in-and-out Norwegian patients using the bifactor analysis [11]. Although the one-factor model obtained most empirical support, two-, three-, and four-factor solutions also emerged in the current literature. A two-factor model was proposed by Ribeiro et al. based on the principal component analysis with varimax rotation on responses from a mixed sample of community-dwelling and clinical old adults [16]. Bendixen et al. found a similar two-factor solution among a sample of elderly with depression, dementia, or psychosis [17]. A three-factor model was first proposed by Márquez-González et al. among 302 old adults living in Spanish communities using principal-components analysis with varimax rotation [18]. Guan also obtained a similar three-factor among 1318 old adults living in Beijing communities with the same method [19]. Finally, a four-factor model was proposed by Diefenbach et al. among a mixed sample of 140 clinical and non-clinical old participants using principal components analysis [20]. These inconsistent findings regarding dimensionality of the GAI can be partly attributed to the analytic methods chosen: Traditional factorial analysis methods such as exploratory factor analysis (EFA) and principal components analysis (PCA) are mainly employed in those studies, and these methods may result in distorted results due to small size and unsatisfied assumptions [21, 22]. More recently, Molde et al. [23] resolved debates about the factor structure of the GAI with bifactor modeling in an extensive dataset with 3731 older adults from 10 national samples and found a primary unidimensional general factor of the GAI across nations.

Mokken scale analysis (MSA), a more sophisticated tool based on nonparametric item response theories, has been proposed to assess dimensionality [24, 25]. It is developed on the basis of the Guttman scaling model, which assumes that scale items are hierarchically ordered along levels of a latent construct. It is less restrictive concerning statistical assumptions and sample size than IRT models, such as Rasch model and logistic models. Compared to traditional factorial analysis, MSA has advantages in conducting dimensionality investigation and model evaluation at the same time, avoiding “difficult factors” and distortions due to item-score distributions. It is a better fit for discrete data sets [22]. The most general Mokken model, monotone homogeneity model (MHM) assumes unidimensionality, local independence, and latent monotonicity [24]. Moreover, the unidimensionality assumption of MHM contributed precisely to test the latent structure of an inventory through automated item selection procedure (AISP) [26, 27]. In a scale formed by Mokken analysis, the sum score of all items can be used as the indicator of the latent trait [24]. It is worth noting that the scale score is ordinal in nature, but it can be interpreted and used as interval values if ordinal transformations have no severe impact on the substantive interpretations of further statistical analyses [28]. Our study would adopt this method to provide complemental evidence to studies on the factor structure of the GAI.

Different groups of people may have different expressions of anxiety and depression. Previous studies indicated that females tended to report more anxiety symptoms than males did [29, 30], but this gender difference disappeared with age increasing [31]. However, before coming to these conclusions, measurement invariance needs to be justified: this instrument must measure the same anxiety symptom of the same extent in all groups [32]. Several researchers have realized the problem. They examined measurement invariance across sex and ages and found no item bias existed [11, 13, 33]. When developing the international translations of the GAI, researchers often have difficulties in finding the exact corresponding words in their languages. For example, the Portuguese version [16], the Spanish version [18], and the Chinese version [34] have different translations of the item “I have butterflies in my stomach” with the original Australia version [35]. In addition, Molde et al. pointed out that due to different understandings of the same item content, even the translation itself implied potential changes in the psychometric properties of the individual item and the whole scale [11]. It is still necessary to examine the item bias of the instrument in different cultures and languages.

Therefore, the present study had two aims: 1) to establish the factor structure of the GAI in a large Chinese sample using Mokken scale analysis [24, 25]; 2) to examine the measurement invariance of the instrument across different groups using DIF analysis.

## Methods

### Data and sample

This is a study of secondary data analysis. The data was drawn from a publicly available dataset, the Chinese National Survey Data Archive (CNSD), which was collected by an extensive survey regarding the mental health of elderly adults [14]. Forty-five communities were randomly selected in Beijing, China, including old communities, new communities, and large villages. Thirty elderly adults in each community were selected by a systematic sampling method. The investigators read the items in the survey one by one, and the participants provided answers corresponding to those items. Finally, a total of 1314 valid records were collected regarding the GAI-CV, 59.5% of which were from females. The age of all participants ranged from 60 to 95 years, with a mean of 71.35 years (SD = 7.45). Other socio-demographic information and clinical characteristics of the sample were presented in Table 1.

**Table 1:** Other socio-demographic and clinical characteristics of the sample

	female	male	total
Marital status			
Married	531 (54%)	445 (46%)	976 (100%)
Unmarried	2 (40%)	3 (60%)	5 (100%)
Divorced	43 (61%)	27 (39%)	70 (100%)
Widowed	206 (78%)	57 (22%)	263 (100%)
Education			
Primary school	179 (67%)	88 (33%)	267 (100%)
middle school	189 (58%)	135 (42%)	324 (100%)
High school	140 (56%)	111 (44%)	251 (100%)
College degree or above	145 (45%)	176 (55%)	321 (100%)
Other	129 (85%)	22 (15%)	151 (100%)
Somatic diseases			
No diseases	110 (50%)	112 (50%)	222 (100%)
At least one diseases	648 (62%)	404 (38%)	1052 (100%)
Total	782 (60%)	532 (40%)	1314 (100%)

### Measures

All participants completed three scales (including the Geriatric Anxiety Inventory-Chinese Version (GAI-CV) and two other scales) and provided information about their sociodemographic characteristics and health status. The other two scales were to measure self-care ability of daily living and social interactions respectively, and would not be the focus of this study.

The GAI-CV is the Chinese version of the Geriatric Anxiety Inventory [3]. It was developed following a standard two-stage procedure of translation and back-translation from the original version [34]. It comprises 20 items (e.g., I worry a lot of the time). Participants are asked to make a dichotomous response to the description of each item (agree/disagree). A sum of these ratings composes a measure of general anxiety symptoms (ranged from 0 to 20), with higher scores indicating more anxiety. The GAI-CV has demonstrated sound psychometric properties in community-dwelling old adults in Beijing. Its internal consistency reliability as Cronbach’s *α* is 0.94. It has a high correlation with scores on the Beck Anxiety Inventory (*r* = 0.60). More detailed information about measures and procedures should be referred to the report of the survey [14].

### Statistical analyses

We conducted a Mokken scale analysis to examine the factor structure (i.e., dimensionality test) using “mokken” package in the statistical software R [24, 25, 36]. The mokken package offers an automated item selection algorithm (aisp) to produce unidimensional subscales from all items. The resulting pattern and scalability of each item (expressed by H_*i*_) signal the structures of the inventory [26, 37]. It also provides procedures to assess the assumptions of local independence and monotonicity [38, 39].

Examining of measurement invariance (i.e., DIF analysis) was proceeded by another package called “difR” in the statistical software R [37]. We applied the logistic regression approach to detect both uniform and nonuniform DIFs [40, 41]. Previous studies have indicated that females were more anxious than males and the elderly who had somatic diseases reported a higher level of anxiety. Therefore, we would focus on the DIF analysis across sex and somatic diseases. More specifically, the following analysis concerned about whether there was any item bias between females and males and between populations who had no disease and those who had at least one kind of somatic disease.

## Results

### Descriptive statistics

Descriptive statistics of the items and the scale for the GAI-CV were presented in Table 2. The endorsement rate for each item is relatively low, all less than 20%.

**Table 2:** Descriptive statistics of the items (upper panel) and the scale (lower panel) for the GAI-CV

Item	M	SD	H_j_	SE	citc
1	0.15	0.35	0.464	0.029	0.541
2	0.16	0.37	0.443	0.030	0.501
3	0.09	0.28	0.591	0.028	0.689
4	0.11	0.32	0.518	0.028	0.631
5	0.08	0.28	0.579	0.030	0.666
6	0.17	0.37	0.585	0.026	0.661
7	0.12	0.33	0.590	0.024	0.723
8	0.18	0.39	0.622	0.026	0.677
9	0.18	0.38	0.641	0.024	0.717
10	0.11	0.31	0.618	0.023	0.753
11	0.13	0.34	0.607	0.023	0.741
12	0.13	0.33	0.429	0.030	0.514
13	0.11	0.32	0.575	0.025	0.704
14	0.07	0.26	0.535	0.035	0.590
15	0.05	0.23	0.630	0.037	0.620
16	0.08	0.27	0.585	0.030	0.671
17	0.04	0.18	0.747	0.033	0.589
18	0.06	0.23	0.477	0.042	0.474
19	0.06	0.24	0.644	0.032	0.664
20	0.11	0.32	0.564	0.025	0.691
M			2.19		
SD			4.18		
H			0.565	0.020	
α			0.937		
λ_2_			0.940		
MS			0.947		

Note. *N = 1314. H*_*j*_ Item-scalability coefficient, *SE* Standard error of item scalability coefficient, *citc* Corrected item–test correlation, *H* Total-scalability coefficient, *α* Cronbach’s alpha, *λ*_*2*_ Guttman’s lambda-2, *MS* Molenaar–Sijtsma method

### Examining factor structure

Scalability coefficients play an essential role in evaluating item quality. The results were also presented in Table 2. The Inter-item scalability coefficients (H_*ij*_), scalability of each item pair in this analysis was always higher than 0.35. The item scalability coefficients, representing the accuracy of item order for respondents on the latent variable based on total scale scores, were more substantial than the suggested lower bound of 0.3 [38, 39], ranged from 0.43 to 0.75. Moreover, the whole inventory had a scalability coefficient H of 0.56, which suggested a scale of strong strength [38, 39]. The 20 items had an excellent internal consistency (Cronbach’s alpha =0.94).

**Fig. 1:**
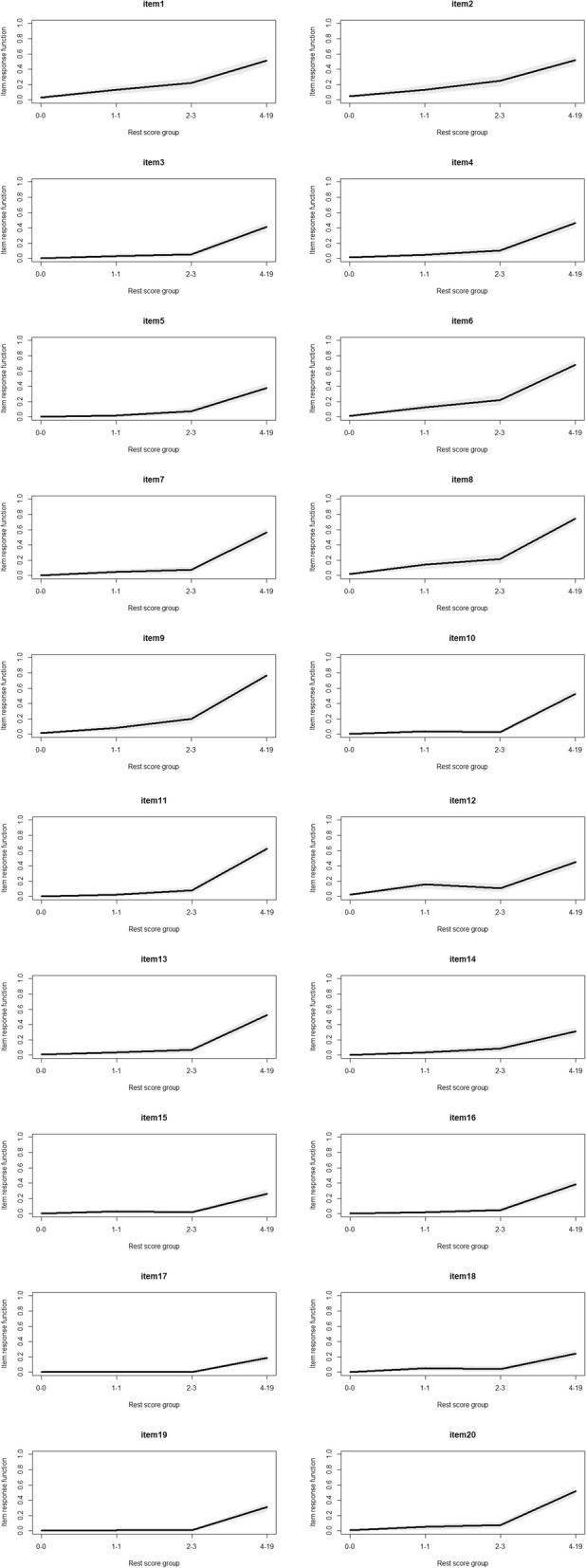
Monotonicity plots of the GAI-CV items

Moreover, local independence and monotonicity were examined to ensure the data were adequately fit to the Mokken scale. For local independence, no item pairs were flagged as locally dependent according to two indices (W1 and W2) calculated in the conditional association procedure [38]. That is, there is no evidence of local dependence. For monotonicity, the results showed that only item 12 violated the monotonicity assumption, but the violation was not significant (See Table 3). Mokken package also provides a simple index called crit for monotonicity seriousness evaluation of each item. It was calculated based on item scalability coefficients H_*i*_, choice frequency, and the magnitude and significance of monotonicity violation. According to a rule of thumb, an item with a crit value less than 40 indicates no serious violation [42]. Item 12 had a crit value of 31, and should not be discarded from the Mokken scale. Graphical analysis indicated that all except Item 12 showed monotonical increases (see Fig. 1). Item 12 showed a significant decrease in the middle, but the impact on its item response function was minimal.

**Table 3:** Output of assessment of monotonicity

Item	#ac	#vi	#zsig	crit
1	6	0	0	0
2	6	0	0	0
3	6	0	0	0
4	6	0	0	0
5	6	0	0	0
6	6	0	0	0
7	6	0	0	0
8	6	0	0	0
9	6	0	0	0
10	6	0	0	0
11	6	0	0	0
12	6	1	0	31
13	6	0	0	0
14	6	0	0	0
15	3	0	0	0
16	6	0	0	0
17	0	0	0	0
18	6	0	0	0
19	6	0	0	0
20	6	0	0	0

Note. *N = 1314.* #ac = number of active pairs that were investigated; #vi = number of violations in which the item is involved; # zsig = number of significant z-values; crit = Crit value

We further investigated the dimensionality for all the 20 items by conducting iterative automated item selection. The results were presented in Table 4. According to the suggestions from Hemker et al., lower bound c started from 0 to 0.75 with increment steps of 0.05. For 0 ≤ c ≤ 0.4, all items were selected in one scale, with H_*i*_ ranging from 0.56 to 0.89. For c = 0.45, one scale with 19 items was formed, and item 12 was dropped due to H < 0.45. For 0.5 ≤ c ≤ 0.55, two items (item 1 and item 2) were dropped out from the original, left one long scale with 16 items and one small short scale with two items (item 12 and item 18). The value of H_*i*_ in the long scale ranged from 0.63 to 0.89; the short scale had the same two H_*i*_ values, 0.76. For c = 0.6, another two items (item 4 and item14) were got unscalable. For 0.6 ≤ c ≤ 0.75, three or four scales formed and the majority of items remained unscalable. For c = 0.8, only two scales (item 9 and item 17; item 8 and item 19) were kept, and for c = 0.85, one scale with two items (item 9 and item 17) remained. Finally, no items passed the automated selection. The results were in accordance with typical patterns of unidimensionality described by Hemker et al., which exhibited a large scale at the beginning and split into several small scales with increasing c. In common practice, the procedure is often implemented for c = 0.3 [25]. Thus, the unidimensionality of the GAI-CV was confirmed.

**Table 4:** The results of automated item selection procedure

*c*	Results	Item Numbers				
		Scale 1	Scale 2	Scale 3	Scale 4	Unscalable
0–0.4	1: 20	1–20				
0.45	1: 19	1–11, 12–20				12
0.5–0.55	2:16, 2	3–11, 13–17, 19–20	12, 18			1, 2
0.6	2:14, 2	3, 5, 6–11, 13, 15–17, 19–20	12, 18			1, 2, 4, 14
0.65	4: 9, 2, 2, 2	8–11, 13, 15–17, 19	12, 18	3, 4	6, 7	1, 2, 5, 20
0.7	3: 7, 3, 2	8–9, 13, 15–17, 19	3, 10, 11	12, 18		1, 2, 4–7, 14, 20
0.75	4: 4, 2, 2, 2	8–9, 17, 19	3, 10	15, 16	12, 18	1, 2, 4-7, 11, 13-14, 20
0.8	2: 2, 2	9, 17	8, 19			1–7, 10–16, 18, 20
0.85	1: 2	9, 17				1–8, 10–16, 18–20

### Examining measurement invariance

**Fig. 2:**
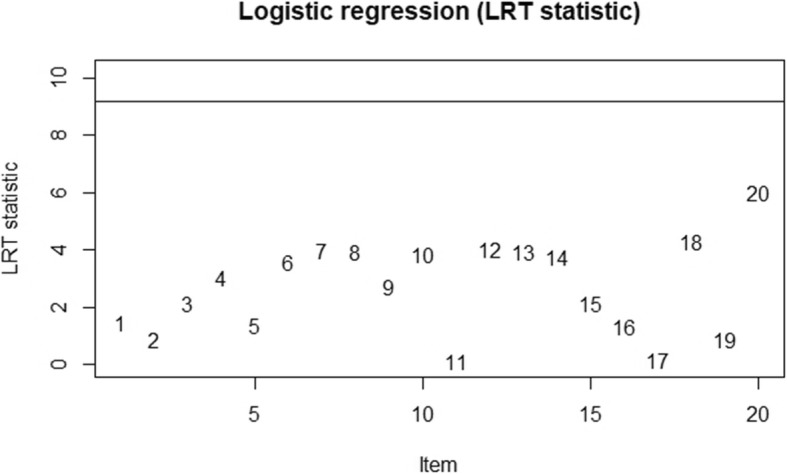
Plots of DIF across sex

**Fig. 3:**
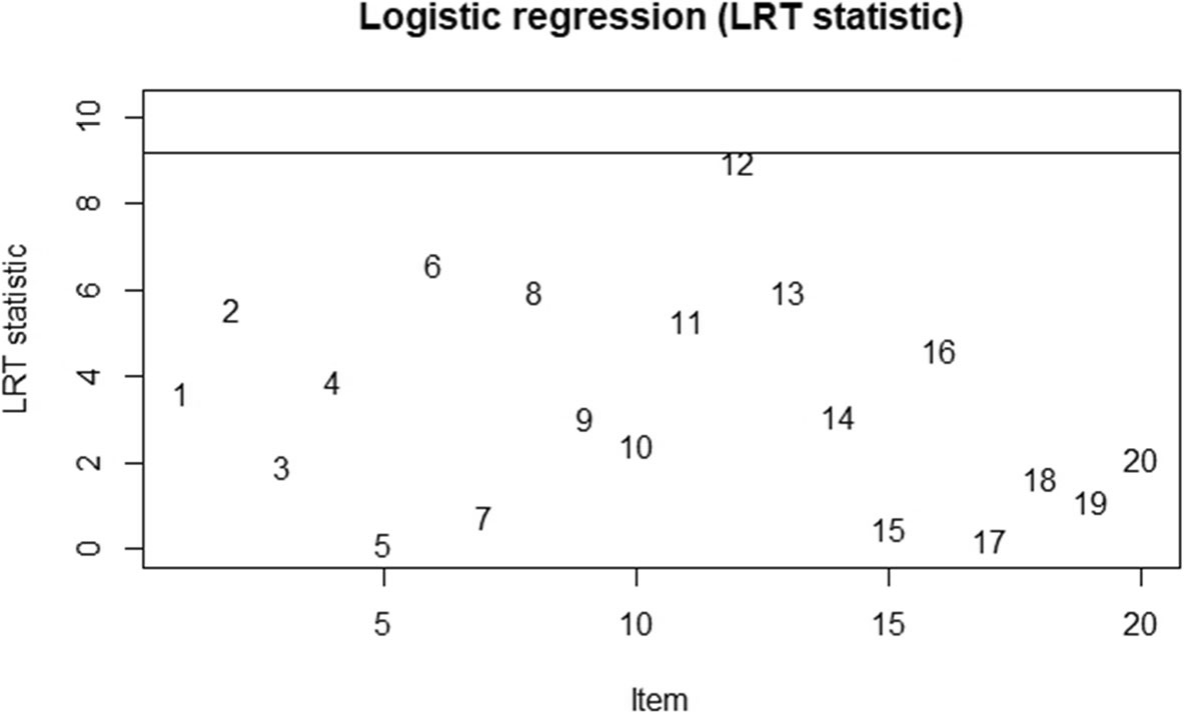
Plots of DIF across disease groups

Following the logistic regression approach, the probability of answering items fitted to the logistic model by the total test score, group membership, and the interaction between these two. We set the significance level of matching criterion at 0.01, and items were detected at the threshold of 9.21. In Table 5, the results of the DIF analysis were exhibited. Regarding sex, item 20 indicated a high logistic regression DIF statistic (Logistic stats = 6.01), which reached significance at a 0.05 level. Nonetheless, the small effect size revealed that the bias was negligible in terms of the measure of R-square. A DIF is considered negligible if R-square ≤ 0. 13, moderate if 0.13 < R-square ≤ 0. 26, and large if R-square > 0.26 [43]. Before detecting item bias between no disease and disease groups, we extracted 269 samples randomly from the disease group for balance. The results indicated that item 6 (Logistic stats = 6.60), item 12 (Logistic stats = 8.97), and item 13 (Logistic stats = 6.00) were significantly different across the subgroups. However, the small effect sizes revealed that these item biases were negligible. Hence, neither uniform nor non-uniform item bias was detected, and the GAI-CV function well across sex and disease groups. These plot outputs were given in Fig. 2 and Fig. 3.

**Table 5:** The results of DIF analysis with logistic regression

Item	Sex				Somatic Disease			
	Statistic	*p*	*R*^2^	Effect size	Statistic	*p*	*R*^2^	Effect size
1	1.46	0.48	0.0015	A	3.66	0.16	0.0105	A
2	0.92	0.63	0.0009	A	5.56	0.06	0.0162	A
3	2.14	0.34	0.0026	A	1.94	0.38	0.0074	A
4	3.06	0.21	0.0034	A	3.92	0.14	0.0118	A
5	1.39	0.50	0.0018	A	0.15	0.93	0.0006	A
6	3.61	0.16	0.0030	A	6.60	0.04^a^	0.0142	A
7	3.98	0.14	0.0038	A	0.76	0.69	0.0021	A
8	3.96	0.14	0.0030	A	5.98	0.05	0.0125	A
9	2.74	0.25	0.0020	A	3.05	0.22	0.0059	A
10	3.86	0.15	0.0039	A	2.44	0.30	0.0071	A
11	0.15	0.93	0.0001	A	5.32	0.07	0.0134	A
12	4.02	0.13	0.0045	A	8.97	0.01^a^	0.0289	A
13	3.97	0.14	0.0040	A	6.00	0.04^a^	0.0184	A
14	3.79	0.15	0.0056	A	3.10	0.21	0.0151	A
15	2.14	0.34	0.0037	A	0.50	0.78	0.0029	A
16	1.36	0.50	0.0018	A	4.61	0.10	0.0173	A
17	0.19	0.91	0.0004	A	0.22	0.90	0.0022	A
18	4.33	0.11	0.0080	A	1.68	0.43	0.0091	A
19	0.89	0.64	0.0014	A	1.13	0.57	0.0052	A
20	6.01	0.04^a^	0.0062	A	2.12	0.35	0.0061	A

*Note.* ^a^indicated significance at 0.05 level. The DIF estimates are classified according to effect size as “A” (negligible effect), “B” (moderate effect), and “C” (large effect”)

## Discussion

The present study reevaluated the psychometric properties of the GAI among a large community-dwelling Chinese elderly sample. Mokken scale analysis was used to determine its dimensionality, and the logistic regression approach was used to detect differential item functions. Results revealed that the Chinese version of the Geriatric Anxiety Inventory possesses sound psychometric properties. It is unidimensional and has no item bias across sex and disease groups.

Previous studies have indicated conflicting findings regarding the factor structure of the GAI. Mainly based on exploratory factor analysis and confirmatory analysis, researchers have proposed one-factor solutions [11–13, 15, 34], two-factor solutions [16, 17], three-factor solutions [18, 19], and a four-factor solution [20]. More recently, Molde et al. [23] addressed the contradictions about the dimensionality of the GAI using bifactor modeling and supported a primarily unidimensional structure across nations. To provide supplementary information about the factor structure debates, we introduced Mokken scale analysis, an NIRT based technique, to determine its dimensionality. Mokken scale analysis provides an effective procedure to determine the factor structure. Other than traditional factor-analytic methods, Mokken scale technique is capable of eliminating effects of the difference in individual item score frequency distributions [44] and provides a clear view on the items’ scalability [22]. Through observing the pattern of AISP, we could differentiate unidimensionality and multidimensionality. The results indicated that the GAI-CV was unidimensional, which supported the conclusion of Yan et al. [34]. Therefore, it is justified to use a simple sum score of the 20 items within the GAI-CV as a reliable index for anxiety among the elderly. It should be noted that the sum score is ordinal in nature, but it can be treated as interval data in case of no serious influence of ordinal transformations on interpretation of further statistical analyses. To our knowledge, this is the first time to explore the GAI with Mokken scale technique. Mokken scale analysis provides a comprehensive output about the scalability of items and the structure of scales [38]. The adoption of Mokken scale analysis in dimensionality test should be recommended in future studies of the GAI in different languages and cultures.

Measurement invariance of the GAI is very important, given researchers often make comparisons among groups with different sex, diseases, and cultures. Only Molde et al. have evaluated the differential item functions across sex, MMSE (The Mini-Mental State Examination) and MADRS (The Montgomery–Asberg Depression Rating Scale) groups. Their results indicated that no item had a substantial bias across those groups. We adopted the logistic regression method, which was one of the most effective and recommended ways to detect DIF [41, 45]. Logistic regression has many advantages over other DIF methods, such as the Mantel Haenszel. It does not require to categorize a continuous criterion variable, and it is capable of modeling both uniform and non-uniform DIF [46]. Previous studies have revealed that females tended to report more anxiety than males, and people with chronic diseases tended to be more anxious than those without somatic diseases. Our study verified that comparisons among those groups were reasonable, and the group differences on the GAI reflected substantial variability rather than differential item functions.

We acknowledged several potential limitations of this study. Although we conducted the analyses in a relative large representative sample, only old adults in Beijing communities were included. Therefore, the generalization of the conclusion to the elderly with various cultural and language backgrounds should be with caution. Future replications in diverse samples in other cultures and languages will be beneficial to the establishment of the worldwide adaptability of the GAI. Besides, our sample did not include clinical patients (e.g., older adults with a primary anxiety disorder). The generalizability of the findings is limited to those who are not clinically diagnosed with anxiety disorders. Future research should attempt to address the limitation of recruiting clinically disordered samples who met the criteria for a primary anxiety disorder.

### Conclusions

This work is among the few studies to examine the factor structure and measurement invariance of the GAI in a large representative sample. Results of Mokken scale analysis confirmed the unidimensionality of the GAI among non-clinical old adults in Beijing communities, and results of DIF analysis ensured the reasonability of comparisons across sex and somatic groups.

## Data Availability

The raw data is publicly available at. https://osf.io/9k5a4/?view_only=a5371241a2474b5fb97016ccd7c80888.

## Abbreviations

AISP: Automated item selection procedure
CFA: Confirmatory factor analysis
DIF: Differential item functioning
EFA: Exploratory factor analysis
GAD: Generalized Anxiety Disorder
GAI: Geriatric Anxiety Inventory
GAI-CV: Geriatric Anxiety Inventory-Chinese Version
MHM: Monotone homogeneity model
MSA: Mokken scale analysis
PCA: Principal components analysis
